# Intraoperative Near-Infrared Spectroscopy Monitoring of Renal Allograft Reperfusion in Kidney Transplant Recipients: A Feasibility and Proof-of-Concept Study

**DOI:** 10.3390/jcm10194292

**Published:** 2021-09-22

**Authors:** Hien Lau, Alberto Jarrin Lopez, Natsuki Eguchi, Akihiro Shimomura, Antoney Ferrey, Ekamol Tantisattamo, Uttam Reddy, Donald Dafoe, Hirohito Ichii

**Affiliations:** 1 Division of Transplantation, Department of Surgery, University of California, Irvine, CA 92868, USA; hlau3@hs.uci.edu (H.L.); ajarrinl@hs.uci.edu (A.J.L.); neguchi@hs.uci.edu (N.E.); shimo-mu@kid.med.osaka-u.ac.jp (A.S.); ddafoe@hs.uci.edu (D.D.); 2 Division of Nephrology, Hypertension and Kidney Transplantation, Department of Medicine, University of California, Irvine, CA 92868, USA; ferreya@hs.uci.edu (A.F.); etantisa@hs.uci.edu (E.T.); ureddy@hs.uci.edu (U.R.)

**Keywords:** clinical research practice, near-infrared spectroscopy, kidney transplantation, initial allograft function, intraoperative, tissue oxygen saturation

## Abstract

Conventional renal function markers are unable to measure renal allograft perfusion intraoperatively, leading to delayed recognition of initial allograft function. A handheld near-infrared spectroscopy (NIRS) device that can provide real-time assessment of renal allograft perfusion by quantifying regional tissue oxygen saturation levels (rSO_2_) was approved by the FDA. This pilot study evaluated the feasibility of intraoperative NIRS monitoring of allograft reperfusion in renal transplant recipients (RTR). Intraoperative renal allograft rSO_2_ and perfusion rates were measured in living (LDRT, *n* = 3) and deceased donor RTR (DDRT, *n* = 4) during the first 50 min post-reperfusion and correlated with renal function markers 30 days post-transplantation. Intraoperative renal allograft rSO_2_ for the DDRT group remained significantly lower than the LDRT group throughout the 50 min. Reperfusion rates were significantly faster in the LDRT group during the first 5 min post-reperfusion but remained stable thereafter in both groups. Intraoperative rSO_2_ were similar among the upper pole, renal hilum, and lower pole, and strongly correlated with allograft function and hemodynamic parameters up to 14 days post-transplantation. NIRS successfully detected differences in intraoperative renal allograft rSO_2_, warranting future studies to evaluate it as an objective method to measure ischemic injury and perfusion for the optimization of preservation/reperfusion protocols and early prediction of allograft function.

## 1. Introduction

Kidney transplantation (KT) is the treatment of choice for patients with end-stage renal disease due to the improved prognosis and quality of life compared to maintenance dialysis [1]. The current supply of donor kidneys has not met the demand for KT [2]. While living donor renal transplants (LDRT) generally have better outcomes, deceased donor kidneys have been the majority organs supplied for transplant in the United States [3]. In comparison to LDRT, deceased donor renal transplants (DDRT) generally have worse allograft function, a higher rate of complications, and longer hospital stay during the early post-transplant period [4,5]. Moreover, slow initial allograft function has been correlated with an increased risk of acute rejection, higher incidence of long-term allograft loss, and worse patient survival [4,6,7,8]. Therefore, prompt evaluation and treatment are crucial to optimize allograft perfusion and minimize ischemic insult, leading to better allograft function.

Up to now, conventional laboratory markers, such as serum creatinine, urine output in donors and cold ischemic time (CIT) are widely used to predict initial allograft function [9]. However, there are very limited reliable intraoperative methods to assess real-time allograft condition, resulting in late detection of slow allograft function and injury during the early post-transplant period [10]. Furthermore, transplant surgeons currently evaluate and predict initial allograft function in the critical early post-transplantation period through the assessment of color, texture, and capillary refill after reperfusion of the allograft, which is highly subjective and varies greatly based on the surgeons’ experience. Due to the severe shortage of organs, marginal kidneys, which are defined as having a higher percent kidney-donor profile index, are increasingly being transplanted. For this reason, the development of a marker for the early evaluation of allograft quality, which can reliably predict slow allograft function, will assist physicians in the adjustment of therapeutic management, and improve allograft function, thus limiting further allograft injury during the immediate posttransplant period [3,11].

Near-infrared spectroscopy (NIRS) devices can measure regional tissue oxygen saturation levels (rSO_2_) and provide real-time assessment of tissue perfusion based on differences in light-absorbing properties of oxygenated and deoxygenated hemoglobin [12]. The perfusion of kidney allografts measured by a NIRS monitor following pediatric KT has recently been shown to be significantly correlated to the current gold standard, Doppler ultrasound [13]. During the 72-h posttransplantation period, continuous renal NIRS measurements were strongly correlated with serum creatinine and eGFR in pediatric KT recipients [14]. Despite the proven benefits, the utilization of NIRS devices to monitor allograft perfusion has not been investigated intraoperatively because of their inconveniently bulky size and in adult KT due to the large skin-to-kidney distance [15]. A wireless, non-invasive, and handheld NIRS device was recently developed and approved by the FDA; however, its use has only been studied in porcine skin flap and bowel models [16,17,18] to our knowledge.

As measuring renal allograft rSO_2_ levels intraoperatively with a handheld NIRS device can circumvent the distance limitation and offer an objective method for the early assessment of allograft reperfusion, this pilot study examined the feasibility of intraoperative NIRS monitoring in KT recipients by comparing intraoperative allograft rSO_2_ levels between LDRT and DDRT and correlating these levels to conventional markers of renal function during the first 30 days after KT.

## 2. Materials and Methods

### 2.1. Patients Eligibility and Study Design

In this prospective study, adults >18 years of age undergoing either LDRT or DDRT were randomly recruited between September 2018 and March 2020 at the University of California, Irvine Medical Center. The study was approved by the research ethics committee at the University of California Irvine Institutional Review Board (protocol # 2018-4395). Written informed consent was obtained from all participants prior to participation. All methods were carried out in accordance with relevant guidelines and regulations. The study was conducted according to the principles of the Declaration of Helsinki and Istanbul.

### 2.2. Near-Infrared Spectroscopy Measurements

Intraoperative monitoring of renal allograft reperfusion was performed using a handheld, non-invasive, and FDA-approved NIRS tissue oximetry sensor device (Intra.Ox, ViOptix, Inc. Newark, CA, USA) that can provide real-time and instantaneous rSO_2_ measurements [16,18]. During the surgical procedure, rSO_2_ levels were measured pre- and post-reperfusion (i.e., removal of vascular clamps) at 3, 5, 10, and 20 min by placing the device directly over three regions of the allograft: upper pole (UP), renal hilum (RH), and lower pole (LP). NIRS measurements were also taken at 30 and 50 min. The mean arterial pressure of all participants was adjusted to approximately 80–90 mmHg. To optimize the accuracy of rSO_2_ measurements and minimize external light interference, bloodstains on the allograft were cleaned with laparotomy sponges, and surgical lights were moved away before each measurement.

### 2.3. Laboratory Measurements

Serum creatinine, blood urea nitrogen (BUN), urine output, systolic blood pressure (BP), and mean arterial pressure (MAP) were measured preoperatively (pre-op) and on postoperative days (POD) 1, 3, 7, 14 and 30 as part of their standard-of-care clinic visits. The estimated glomerular filtration rate (eGFR) was calculated as previously described [19].

### 2.4. Statistical Analysis

Data normality was analyzed using a Shapiro–Wilk test. Normally distributed continuous variables, including age, weight, height, CIT, length of hospital stay, rSO_2_ levels, systolic BP, and MAP, are expressed as mean ± standard error of the mean (SEM). Median and range are used to present continuous variables with a non-normal distribution. The categorical variable, gender, was described as percentages and analyzed using a chi-square test. An unpaired two-tailed *t*-test was used to compare continuous variables with a normal distribution, while non-normally distributed continuous variables were compared using a two-sided Mann-Whitney U test. The differences in rSO_2_ levels among three regions were analyzed in both groups using a one-way analysis of variance (ANOVA) with Bonferroni correction for multiple comparisons. Linear regression analysis was performed to construct trend lines and estimate the rates of change in rSO_2_ levels from pre-reperfusion to 5 min post-reperfusion and from 20 to 50 min post-reperfusion. Comparisons of rates of change in rSO_2_ levels per minute between two groups were conducted using an analysis of covariance (ANCOVA). The average rSO_2_ levels post-reperfusion at 3, 5, 10, and 20 min were correlated to conventional markers of renal function, including serum creatinine, eGFR, BUN, and urine output, using a non-parametric Spearman’s correlation coefficient test and to hemodynamic parameters related to allograft perfusion, including systolic BP and MAP, using a parametric Pearson’s correlation coefficient test. A *p* < 0.05 was defined as statistically significant. All statistical analyses were performed using SPSS Statistics 25 (IBM SPSS, Chicago, IL, USA).

## 3. Results

### 3.1. Characteristics of Living and Deceased Renal Transplant Recipients

Of the seven KT recipients randomly recruited, three recipients underwent LDRT and four recipients received DDRT. Both groups had no differences in age, gender, weight, and height (Table 1). There was no significant difference in the length of hospital stay between the two groups (LDRT: 6.3 ± 1.3 days vs. DDRT: 7.5 ± 1.2 days; *p* = 0.545) (Table 1). The average CIT were 2.2 ± 0.1 h in LDRT and 25.3 ± 1.4 h in DDRT (*p* = 0.057) (Table 1).

### 3.2. Tissue Oxygen Saturation (rSO_2_) Level at the UP, RH, and LP of the Kidney Allograft in Living and Deceased Donor Renal Transplants after Reperfusion

**Figure 1:**
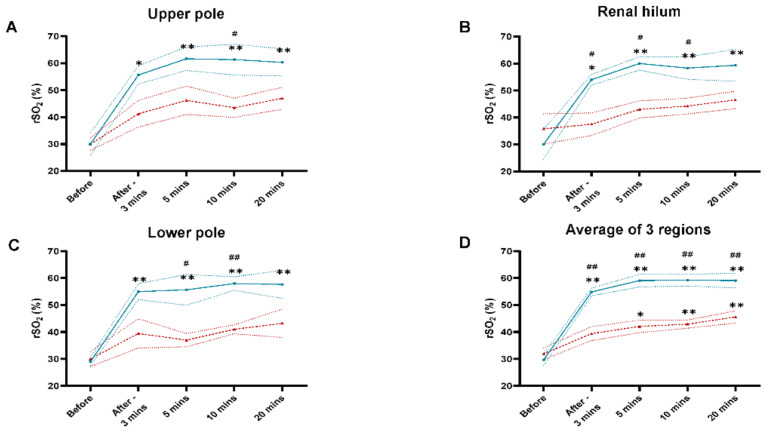
Tissue oxygen saturation (rSO_2_) level curves of the kidney allograft in living (*n* = 3, solid turquoise line and closed circles) and deceased donor renal transplants (*n* = 4, dash red line and closed triangles) before and after 20 min (mins) of reperfusion. Tissue oxygen saturation levels of the kidney allograft in living and deceased donor renal transplants were measured at three regions (upper pole, renal hilum, and lower pole) using a handheld tissue oximetry sensor device before and after reperfusion at 3, 5, 10 and 20 min. (**A**) Tissue oxygen saturation level curves measured at the upper pole of the kidney allograft. (**B**) Tissue oxygen saturation level curves measured at the renal hilum of the kidney allograft. (**C**) Tissue oxygen saturation level curves measured at the lower pole of the kidney allograft. (**D**) Average tissue oxygen saturation level curves measured at three regions of the kidney allograft. * *p* < 0.05 vs. before reperfusion. ** *p* < 0.01 vs. before reperfusion. ^#^ *p* < 0.05 vs. deceased donor kidney allografts. ^##^ *p* < 0.01 vs. deceased donor kidney allografts. Data are expressed as mean ± SEM.

Allograft rSO_2_ levels pre-reperfusion in two groups were not different at UP, RH, and LP (LDRT: 30.0 ± 4.2%, 30.0 ± 5.5%, 29.0 ± 1.5% vs. DDRT: 30.0 ± 2.3%, 35.8 ± 5.6%, 30.0 ± 2.8%, respectively) (Figure 1A–C). After the removal of vascular clamps, rSO_2_ levels at UP of the allograft in LDRT group were significantly higher than DDRT group at 10 min, but not at 3, 5, or 20 min (LDRT: 61.3 ± 5.7%, 55.7 ± 3.4%, 61.7 ± 4.4%, 60.3 ± 5.0% vs. DDRT: 43.5 ± 3.6%, 41.3 ± 5.0%, 46.3 ± 5.3%, 47.0 ± 4.1%, respectively) (Figure 1A–C). At RH, rSO_2_ levels in LDRT group were significantly higher than DDRT group at 3, 5, and 10 min after reperfusion, but not at 20 min (LDRT: 54.0 ± 2.0%, 60.0 ± 2.5%, 58.3 ± 4.2%, 59.3 ± 5.9% vs. DDRT: 37.5 ± 4.2%, 43.0 ± 3.2%, 44.3 ± 3.0%, 46.5 ± 3.2, respectively) (Figure 1A–C). When rSO_2_ levels was measured at LP, LDRT group had significantly greater measurements than DDRT group at 5 and 10 min, but not at 3 and 20 min (LDRT: 55.7 ± 5.7%, 58.0 ± 2.5%, 55.0 ± 3.0%, 57.7 ± 5.2% vs. DDRT: 37.0 ± 2.5%, 41.0 ± 1.7%, 39.5 ± 5.4%, 43.3 ± 5.3%, respectively) (Figure 1A–C). Although the average rSO_2_ levels at three regions were similar between two groups pre-reperfusion, these levels became significantly higher in LDRT group at 3-, 5-, 10-, and 20-min post-reperfusion (LDRT: 29.7 ± 2.1%, 54.9 ± 1.5%, 59.1 ± 2.4%, 59.2 ± 2.2%, 59.1 ± 2.7% vs. DDRT: 32.0 ± 2.2%, 39.4 ± 2.6%, 42.1 ± 2.3%, 42.9 ± 1.6%, 45.6 ± 2.3%, respectively) (Figure 1D). Even after 30- and 50-min post-reperfusion, LDRT group had significantly greater average rSO_2_ levels at three regions (LDRT: 66.3 ± 2.0%, 63.2 ± 3.2% vs. DDRT: 46.0 ± 3.2%, 43.0 ± 4.6%, respectively) (Supplementary Figure S1A).

In the LDRT group, rSO_2_ levels measured at 3-, 5-, 10-, and 20-min post-reperfusion were significantly increased compared to the levels pre-reperfusion at all three regions (Figure 1A–C). However, these differences were not observed in the DDRT group as changes in rSO_2_ levels at all three regions from pre-reperfusion to post-reperfusion at 3, 5, 10, and 20 min were not significant (Figure 1A–C). The average rSO_2_ levels at three regions in the LDRT group were significantly greater post-reperfusion at 3, 5, 10, and 20 min compared to pre-reperfusion (Figure 1D). In the DDRT group, changes in rSO_2_ levels from pre-reperfusion to post-reperfusion only reached statistical significance at 5, 10, and 20 min, but not at 3 min (Figure 1D). Moreover, the average rSO_2_ levels at three regions in both groups had no significant changes from 5 to 50 min post-reperfusion (Supplementary Figure S1A). 

To determine whether rSO_2_ levels were higher at a particular region of the allograft, a comparison of measurements among three regions was performed and showed no significant differences at all-time points in both groups (Table 2).

### 3.3. Linear Regression Analysis of Tissue Oxygen Saturation (rSO_2_) Levels of the Kidney Allograft in Living and Deceased Donor Renal Transplant after Reperfusion

**Figure 2:**
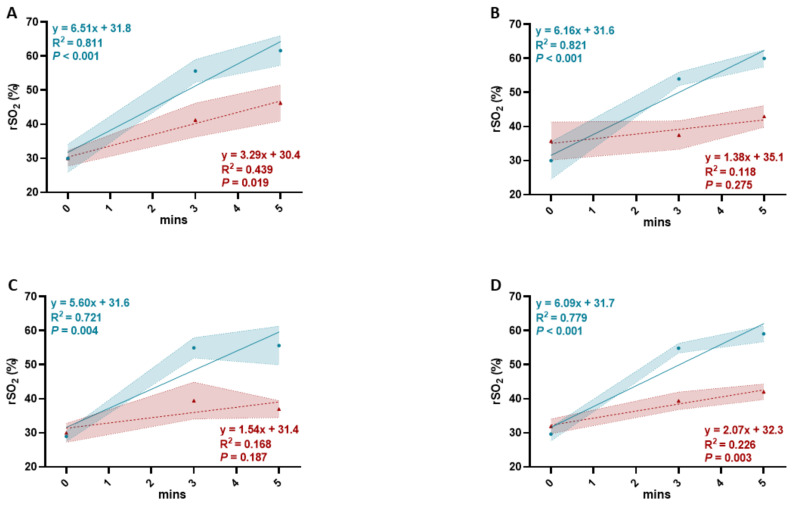
Linear regression analysis of tissue oxygen saturation (rSO_2_) levels of the kidney allograft in living (*n* = 3, solid turquoise line and closed circles) and deceased donor renal transplants (*n* = 4, dash red line and closed triangles) over 5 min (mins) after reperfusion. Trend lines and R^2^ coefficients of tissue oxygen saturation levels measured at three regions (upper pole, renal hilum, and lower pole) of the kidney allograft over 5 min of reperfusion in living and deceased donor renal transplants were calculated using linear regression analysis. (**A**) Trend lines and R^2^ coefficients of tissue oxygen saturation levels measured at the upper pole of the kidney allograft. (**B**) Trend lines and R^2^ coefficients of tissue oxygen saturation levels measured at the renal hilum of the kidney allograft. (**C**) Trend lines and R^2^ coefficients of tissue oxygen saturation levels measured at the lower pole of the kidney allograft. (**D**) Trend lines and R^2^ coefficients of average tissue oxygen saturation levels measured at three regions of the kidney allograft. Trend line equations, R^2^ coefficients, and *p*-values for living and deceased donor renal transplants are displayed in matching colors. Data are expressed as mean ± SEM.

In the DDRT group, the increase in average rSO_2_ levels at three regions only achieved statistical significance after 5 min of reperfusion compared to 3 min in the LDRT group (Figure 1D). This indicated a slower reperfusion rate in the DDRT group; therefore, linear regression analysis of the increase in rSO_2_ levels over 5 min of reperfusion was performed to compare the rates of increase in rSO_2_ levels between two groups at all three regions (Figure 2A–D). While all trend lines of the increase in rSO_2_ levels with time were positive, only three regions of the kidney allograft in LDRT group and UP of the allograft in DDRT group reached statistical significance (UP: *p* < 0.001, =0.019; RH: *p* < 0.001, =0.275; LP: *p* = 0.004, 0.187, respectively) (Figure 2A–C). Significantly positive trend lines of the increase in average rSO_2_ levels at three regions over 5 min of reperfusion were observed in both groups (*p* < 0.001, = 0.003, respectively) (Figure 2D). A comparison of slopes of the trend lines revealed that the LDRT group had 1.98, 4.46-, and 3.63-times higher rates of increase in rSO_2_ levels with time at UP, RH, and LP compared to the DDRT group, respectively (Table 3). Moreover, the rate of increase in the average rSO_2_ levels at three regions in the LDRT group was 2.94 times higher than the DDRT group after 5 min of reperfusion (Table 3). Linear regression analysis further revealed that rates of change in average rSO_2_ levels at three regions from 5 to 50 min post-reperfusion did not significantly differ from zero in both groups and were similar between these two groups (0.126%·min^−1^, 0.034%·min^−1^; *p* = 0.103, 0.673, 0.431, respectively) (Supplementary Figure S1B). 

### 3.4. Correlation of Averaged Tissue Oxygen Saturation (rSO_2_) Levels Measured at UP, RH, LP of Kidney Allograft with Renal Function and Hemodynamic Parameters of Living and Deceased Donor Renal Transplants before and after Transplantation

**Figure 3:**
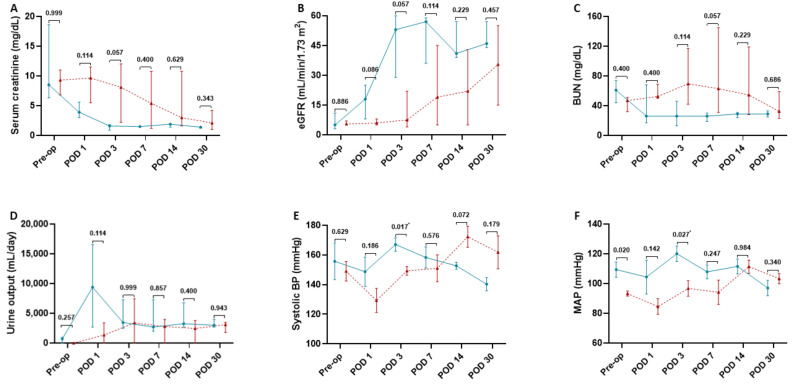
Renal function and hemodynamic parameters of living (*n* = 3, solid turquoise line and closed circles) and deceased donor renal transplants (*n* = 4, dash red line and closed triangles) before and after transplantation. Renal function and hemodynamic parameters of living and deceased donor renal transplants were measured preoperatively (pre-op) and on postoperative day (POD) 1, 3, 7, 14 and 30. (**A**) Serum creatinine levels. (**B**) Estimated glomerular filtration rate (eGFR). (**C**) Blood urea nitrogen levels (BUN). (**D**) Urine output. (**E**) Systolic blood pressure (BP). (**F**) Mean arterial pressure (MAP). The number above each time point represents the *p*-value of living vs. deceased donor renal transplants. * *p* < 0.05. Serum creatinine levels, eGFR, and BUN levels are expressed as median and range. Systolic BP and MAP are expressed as mean ± SEM.

A comparison of conventional markers related to renal function, including serum creatinine, eGFR, BUN, and urine output, on the pre-op day and POD 1, 3, 7, 14, and 30 indicated no significant differences between the two groups (Figure 3A–D). Hemodynamic parameters, including systolic BP and MAP, of the LDRT group, only became significantly higher than the DDRT group on POD 3 and were similar on the pre-op day as well as POD 1, 7, 14, and 30 (Figure 3E,F). Correlation analysis showed average rSO_2_ levels at three regions correlated well with markers of renal function from POD 1 to 14 (Table 4). At 5 min post-reperfusion, average rSO_2_ levels were significantly associated with a decrease in serum creatinine on POD 1 and 3 (*r* = −0.93, −0.96, respectively), an increase in eGFR on POD 1, 3, and 7 (*r* = 0.93, 0.96, 0.89, respectively), a decrease in BUN on POD 3 and 7 (*r* = −0.86, −0.93, respectively), and an increase in urine output on POD 1 (*r* = 0.82) (Table 4). Average rSO_2_ levels at 10 min also showed a strong negative correlation with serum creatinine on POD 3 (*r* = −0.89), positive correlation with eGFR on POD 3 (*r* = 0.89), and negative correlation with BUN on POD 7 (*r* = −0.86). After 20 min of reperfusion, average rSO_2_ levels were significantly correlated with a decrease in serum creatinine on POD 3 (*r* = −0.86), an increase in eGFR on POD 3, 7, and 14 (*r* = 0.86, 0.86, 0.89, respectively), a decrease in BUN on POD 3 and 7 (*r* = −0.96, −0.93, respectively), and an increase in urine output on POD 14 (*r* = 0.79). When correlated to hemodynamic parameters, average rSO_2_ levels at three regions over 20 min of reperfusion showed a strong association from POD 1 to 14 (Table 4). At 3 min of reperfusion, average rSO_2_ levels were significantly correlated with the increases in systolic BP and MAP on POD 1 (*r* = 0.88, 0.83) (Table 4). After 5 and 10 min of reperfusion, average rSO_2_ levels were strongly associated with the increases in systolic BP and MAP on POD 3 (*r* = 0.84, 0.84, 0.87, 0.76, respectively) (Table 4). Moreover, average rSO_2_ levels at 5- and 10-min post-reperfusion showed a strong negative correlation with systolic BP on POD 14 (5 min: *r* = −0.79, −0.79, respectively) (Table 4). At 20 min post-reperfusion, average rSO_2_ levels were significantly associated with the increase in MAP on POD 3 (*r* = 0.85). After 30 days of KT, rSO_2_ levels at all-time points were not significantly associated with any markers of renal function or hemodynamic parameters measured in the current study (Table 4).

## 4. Discussion

Current clinical and laboratory indices are inadequate for the early detection of slow kidney allograft function and injury during the immediate post-transplant period [20]. NIRS monitoring has been demonstrated to be an earlier predictor of acute kidney injury (AKI) after cardiac surgery, and, recently, used to measure the early postoperative allograft perfusion status in pediatric KT recipients [13,14,21]. However, no studies have explored the intraoperative application of NIRS monitoring in KT. The current pilot study examined the feasibility of intraoperative NIRS monitoring of kidney allografts by comparing intraoperative rSO_2_ levels between LDRT and DDRT and correlating these levels to conventional markers of renal function in the first 30 days after KT. The major finding was that allografts from the LDRT recipients had significantly higher rSO_2_ levels and faster rates of increase in rSO_2_ levels than the DDRT recipients after reperfusion. Moreover, intraoperative rSO_2_ levels were strongly correlated with renal function and hemodynamic parameters up until POD 14.

To our knowledge, this is the first study to establish a baseline renal allograft rSO_2_ level measured intraoperatively with a handheld NIRS device, which was approximately 30% pre-reperfusion in both the LDRT and the DDRT recipients. This baseline value agrees with previous studies, concluding that a rSO_2_ level of 30% or less represents significant free flap ischemia and requires operative correction [16,22]. The validity of this baseline value was further confirmed by the similarity between rSO_2_ levels before reperfusion of KT recipients. Our results of the increase in rSO_2_ levels post-reperfusion in both groups support others’ observation that rSO_2_ levels of rat kidneys with short and long CIT rose immediately post-reperfusion [23]. The novel findings that kidney allografts from LDRT had significantly higher intraoperative rSO_2_ levels than DDRT post-reperfusion was expected since deceased donor kidney allografts generally suffered from prolonged ischemic injury and decreased microvascular flow [24]. This impairment in microvascular perfusion has been attributed to a 42% and 16% reduction of total blood flow volume and endothelial glycocalyx thickness in the peritubular capillary network [25]. Our data are in accordance with previous findings that kidneys with acute injury had significantly worse rSO_2_ levels during cardiac surgery than those without acute injury [26]. Interestingly, Vidal et al. have shown renal allograft rSO_2_ levels from POD 1 to 3 were comparable between pediatric LDRT and DDRT [14]. Furthermore, the increase in rSO_2_ levels from POD 1 to 3 was not significantly different in recipients with or without delayed allograft function (DGF), indicating that the renal perfusion status was similar between two groups during the early postoperative course despite differences in kidney function [14]. These results suggest the slow initial allograft function is most likely due to the ischemic damage that occurred during organ preservation and reperfusion. As a recent study has reported a 20% decline in kidney rSO_2_ levels from the baseline could predict hypoperfusion and AKI, a more than 20% difference in rSO_2_ levels between LDRT and DDRT groups from 30 to 50 min post-reperfusion indicated that allografts in DDRT recipients were markedly under perfused [21]. Additionally, the immediate postoperative rSO_2_ level has previously been demonstrated to be approximately 70% in kidney allografts of both pediatric LDRT and DDRT, including recipients with DGF [14]. Using this value as the baseline, the rSO_2_ level of approximately 45% at 50 min after reperfusion in DDRT recipients represents substantial hypoperfusion and inadequate reperfusion capacity. This could explain why half of DDRT recipients in the current study experienced slow allograft function (serum creatinine > 1.5 mg/dL and creatinine reduction ratio < 20% between POD 1 and 3) while all LDRT recipients had immediate allograft function [7]. Intraoperative assessment of allograft reperfusion may offer a better objective method to evaluate the extent of ischemic injury and initial allograft function.

Allografts from LDRT recipients were observed to have faster rates of increase in rSO_2_ levels up until 5 min post-reperfusion compared to DDRT recipients. This finding supports our observation that allografts in DDRT recipients required more time to reach higher rSO_2_ levels post-reperfusion. In accordance with these results, rat kidneys with a longer CIT have been shown to have a slower rate of reperfusion compared to a shorter CIT [23]. Our findings that rSO_2_ levels did not change significantly from 5 to 50 min and the rates of change in rSO_2_ levels remained stable after 5 min of reperfusion is in line with previous studies [23,27]. Vaughan et al. has demonstrated that a sharp increase in rSO_2_ levels during the first 10 min post-reperfusion in rats with a 45-min ischemia was followed by a flat rate of change in rSO_2_ levels from 10 min to 4.5 h post-reperfusion [23]. Grosenick et al. have reported that rSO_2_ levels in rat kidneys rose quickly within 3 min post-reperfusion and stayed unchanged until the end of the experiment [27]. The decrease in perfusion rates after the first few minutes of reperfusion could be because allografts from the LDRT recipients had significantly larger blood vessel diameter and higher microvascular blood flow velocity than the DDRT recipients at 5 min but not at 30 min after reperfusion [25]. As the reproducibility of NIRS monitoring has been proposed to be improved by at least two simultaneous measurements, our study found no differences in rSO_2_ levels between the three regions in both groups [28]. Similarly, a previous study has shown postoperative rSO_2_ levels of pediatric KT recipients were similar at the upper and lower poles [13]. Taken together, these findings confirm the responsiveness and validity of intraoperative NIRS monitoring to quantify changes in allograft reperfusion during the early reperfusion period.

In various clinical studies, NIRS monitoring has been reported to be an earlier indicator of renal hypoperfusion and acute injury compared to conventional makers of renal function [21,26,29]. Our findings of no significant differences in renal function measured between the LDRT and the DDRT recipients, except for higher intraoperative rSO_2_ levels in the former group, are consistent with previous evidence, showing that infants who developed AKI from day 2 to 7 of life had significantly higher rSO_2_ levels, but not serum creatinine and urine output, during the first 24 h of life compared to those without AKI [29]. Another study has reported intraoperative NIRS monitoring was an earlier predictor of AKI after pediatric cardiopulmonary bypass surgery than cystatin C and neutrophil gelatinase-associated lipocalin, which have been demonstrated to increase before significant changes in serum creatinine can be detected [26,30,31]. These findings are plausible as serum creatinine remains within the normal range until 50% of renal function is lost [32]. In concordance with the current results that indicated a strong association of intraoperative rSO_2_ levels with renal function and hemodynamic indices, allograft rSO_2_ levels have been correlated with serum creatinine, eGFR, urine output, and systolic BP during the early postoperative period [13,14,29,33]. As a recent study has utilized renal NIRS measurements to adjust fluid therapy in neonatal digestive surgeries, our findings strengthen the growing evidence that NIRS monitoring of allograft perfusion will assist to improve the current initial post-transplant management [34]. Although our pilot study demonstrated several significant correlations between intraoperative rSO_2_ level with conventional markers of renal function, post-transplant urine output remains the most common biomarker that indicates improvement in allograft function at the immediate post-transplant period. Therefore, intraoperative rSO_2_ level at 5 min post-reperfusion may assist clinicians to predict the signs of regaining early allograft function and appropriately modify volume management.

The limitation of our study is its small sample size, which did not allow sensitivity analysis by stratifying DDRT recipients based on CIT, warm ischemic time, the status of initial allograft function, death status, and preservation with or without machine perfusion because all these factors can impact reperfusion capacity and allograft outcomes [6,35,36,37,38]. Even though differences in intraoperative rSO_2_ levels and reperfusion rates between two groups were statistically significant, evaluating intraoperative rSO_2_ levels of allografts with varying severity of the ischemic injury will further validate intraoperative NIRS monitoring and identify cutoff values for the earlier prediction of initial allograft function. Since it is not yet feasible to reliably assess renal allograft rSO_2_ levels in adult recipients after closure due to the large skin-to-kidney distance, the time required for rSO_2_ levels of allografts from DDRT recipients to return to levels that are comparable to those of LDRT recipients could not be determined [15]. As variations in fluid therapy strategies have been shown to affect renal rSO_2_ levels, changes in intraoperative rSO_2_ levels due to different fluid and pharmacologic treatments were also not recorded [34].

In conclusion, this pilot study, to our best knowledge, is the first to show the feasibility of measuring renal allograft rSO_2_ levels intraoperatively in KT recipients with a handheld NIRS device. Intraoperative NIRS monitoring was capable of detecting higher rSO_2_ levels throughout 50 min of reperfusion and faster perfusion rates during the early reperfusion phase in kidney allografts of LDRT recipients compared to those of DDRT recipients. These values were similar between the three regions and strongly associated with conventional markers of renal function up to 14 days after transplantation. Since utilizing a handheld NIRS device offers the advantage of being able to measure renal allograft perfusion by direct contact immediately after reperfusion, future studies will evaluate its intraoperative use as an objective method to assess the ischemic injury and reperfusion capacity for the optimization of preservation/reperfusion protocols and early prediction of initial allograft function.

## Figures and Tables

**Table 1:** Characteristics of living and deceased donor renal transplant recipients.

	LDRT (*n* = 3)	DDRT (*n* = 4)	*p*-Value
**Age (years)**	38.0 ± 9.2	59.5 ± 3.5	0.057
**Female, *n* (%)**	1 (33.3%)	1 (25.0%)	0.999
**Weight (kg)**	69.2 ± 9.5	87.2 ± 7.2	0.183
**Height (m)**	1.6 ± 0.06	1.7 ± 0.01	0.761
**Cold ischemic time (hours)**	2.2 ± 0.1	25.3 ± 1.4	0.057
**Hospital stay (days)**	6.3 ± 1.3	7.5 ± 1.2	0.545

Data are expressed as mean ± standard error of the mean. LDRT: living donor renal transplant. DDRT: deceased donor renal transplant. ICU: intensive care unit.

**Table 2:** Comparison of tissue oxygen saturation (rSO_2_) levels measured at three regions (upper pole, renal hilum, and lower pole) of the kidney allograft in either living or deceased donor renal transplants before and after reperfusion at 3, 5, 10 and 20 min.

	LDRT (*n* = 3)					DDRT (*n* = 4)				
	Before	After-3 min	5 min	10 min	20 min	Before	After-3 min	5 min	10 min	20 min
Upper pole (%)	30.0 ± 4.2	55.7 ± 3.4	61.7 ± 4.4	61.3 ± 5.7	60.3 ± 5.0	30.0 ± 2.3	41.3 ± 5.0	46.3 ± 5.3	43.5 ± 3.6	47.0 ± 4.1
Renal hilum (%)	30.0 ± 5.5	54.0 ± 2.0	60.0 ± 2.5	58.3 ± 4.2	59.3 ± 5.9	35.8 ± 5.6	37.5 ± 4.2	43.0 ± 3.2	44.3 ± 3.0	46.5 ± 3.2
Lower pole (%)	29.0 ± 1.5	55.0 ± 3.0	55.7 ± 5.7	58.0 ± 2.5	57.7 ± 5.2	30.0 ± 2.8	39.5 ± 5.4	37.0 ± 2.5	41.0 ± 1.7	43.3 ± 5.3
*p*-value	0.980	0.918	0.631	0.840	0.940	0.503	0.865	0.275	0.711	0.801

Data are expressed as mean ± SEM. LDRT: living donor renal transplant. DDRT: deceased donor renal transplant. mins: minutes.

**Table 3:** Comparison of the rates of change in tissue oxygen saturation (rSO_2_) levels per minute at three regions (upper pole, renal hilum, and lower pole) of the kidney allograft calculated from linear regression analysis over 5 min of reperfusion in living and deceased donor renal transplants.

	LDRT [95% CI]	DDRT [95% CI]	*p*-Value
**Upper pole (%·min^−1^)**	6.51 [3.70, 9.32]	3.29 [0.668, 5.91]	0.077
**Renal hilum (%·min^−1^)**	6.16 [3.58, 8.73]	1.38 [−1.29, 4.05]	0.011
**Lower pole (%·min^−1^)**	5.60 [2.49, 8.71]	1.54 [−0.879, 3.96]	0.028
**Average of three regions (%·min^−1^)**	6.09 [4.75, 7.42]	2.07 [0.734, 3.41]	<0.001

LDRT: living donor renal transplant. DDRT: deceased donor renal transplant. CI: confidence interval. %·min^−1^: the change in the percentage of tissue oxygen saturation levels per minute.

**Table 4:** Correlation of averaged tissue oxygen saturation (rSO_2_) levels measured at three regions (upper pole, renal hilum, and lower pole) of the kidney allograft with renal function and hemodynamic parameters before and after renal transplants.

		Average rSO_2_ of 3 Regions After							
		3 min		5 min		10 min		20 min	
		*r*	*p*-Value	*r*	*p*-Value	*r*	*p*-Value	*r*	*p*-Value
**Serum Creatinine (mg/dL)**	**POD 1**	−0.57	0.180	−0.93 **	0.003	−0.71	0.071	−0.64	0.119
	**POD 3**	−0.71	0.071	−0.96 **	<0.001	−0.89 **	0.007	−0.86 *	0.014
	**POD 7**	−0.37	0.413	−0.70	0.077	−0.70	0.077	−0.52	0.233
	**POD 14**	−0.14	0.760	−0.39	0.383	−0.68	0.094	−0.36	0.432
	**POD 30**	−0.31	0.504	−0.63	0.129	−0.74	0.058	−0.41	0.355
**eGFR (mL/min/1.73 m^2^)**	**POD 1**	0.66	0.111	0.93 **	0.003	0.75	0.054	0.63	0.139
	**POD 3**	0.71	0.071	0.96 **	<0.001	0.89 **	0.007	0.86 *	0.014
	**POD 7**	0.71	0.071	0.89 **	0.007	0.75	0.052	0.86 *	0.014
	**POD 14**	0.71	0.071	0.75	0.052	0.54	0.215	0.89 **	0.007
	**POD 30**	0.43	0.333	0.69	0.090	0.51	0.248	0.52	0.229
**BUN (mg/dL)**	**POD 1**	−0.29	0.535	−0.64	0.119	−0.36	0.432	−0.54	0.215
	**POD 3**	−0.68	0.094	−0.86 *	0.014	−0.71	0.071	−0.96 **	<0.001
	**POD 7**	−0.75	0.052	−0.93 **	0.003	−0.86^*^	0.014	−0.93 **	0.003
	**POD 14**	−0.43	0.337	−0.61	0.148	−0.75	0.052	−0.61	0.148
	**POD 30**	−0.52	0.229	−0.22	0.641	−0.11	0.818	−0.51	0.248
**Urine Output (mL/day)**	**POD 1**	0.75	0.052	0.82 *	0.023	0.68	0.094	0.75	0.052
	**POD 3**	0.21	0.645	0.29	0.535	0.14	0.760	0.68	0.094
	**POD 7**	0.29	0.535	0.43	0.337	0.29	0.535	0.68	0.094
	**POD 14**	0.75	0.052	0.50	0.253	0.21	0.645	0.79 *	0.036
	**POD 30**	0.36	0.427	0.02	0.969	−0.23	0.613	0.16	0.728
**Systolic BP (mmHg)**	**POD 1**	0.88 **	0.009	0.49	0.261	0.24	0.611	0.53	0.218
	**POD 3**	0.42	0.343	0.84 *	0.018	0.87 *	0.011	0.47	0.288
	**POD 7**	0.50	0.259	0.15	0.743	0.18	0.704	0.12	0.805
	**POD 14**	−0.26	0.569	−0.79 *	0.034	−0.79 *	0.034	−0.65	0.115
	**POD 30**	−0.54	0.209	−0.52	0.229	−0.50	0.256	−0.45	0.317
**MAP (mmHg)**	**POD 1**	0.83 *	0.020	0.57	0.181	0.36	0.425	0.68	0.094
	**POD 3**	0.65	0.117	0.84 *	0.019	0.76 *	0.046	0.85 *	0.015
	**POD 7**	0.65	0.114	0.42	0.350	0.47	0.290	0.47	0.286
	**POD 14**	0.33	0.468	0.07	0.887	−0.11	0.815	0.47	0.289
	**POD 30**	−0.23	0.615	−0.37	0.411	−0.30	0.510	0.08	0.858

eGFR: estimated glomerular filtration rate. BUN: blood urea nitrogen. BP: blood pressure. MAP: mean arterial pressure. POD: postoperative day. * *p* < 0.05. ** *p* < 0.01.

## Data Availability

The data that support the findings of this study are available from the corresponding author upon request.

## Supplementary Materials

The following are available online at https://www.mdpi.com/article/10.3390/jcm10194292/s1, Figure S1: Curves and linear regression analysis of average tissue oxygen saturation (rSO_2_) levels measured at three regions (upper pole, renal hilum, and lower pole) of the kidney allograft in living (solid turquoise line and closed circles, LDRT) and deceased donor renal transplants (dash red line and closed triangles, DDRT) from 5 min to 50 min (mins) after reperfusion.

## Abbreviations

AKI	acute kidney injury
BP	blood pressure
BUN	blood urea nitrogen
CIT	cold ischemic time
DDRT	deceased donor renal transplant
eGFR	estimated glomerular filtration rate
KT	kidney transplant
LDRT	living donor renal transplant
LP	lower pole
MAP	mean arterial pressure
NIRS	near-infrared spectroscopy
POD	postoperative day
RH	renal hilum
rSO_2_	regional tissue oxygen saturation
UP	upper pole
