# Supplementary material for: Intraoperative Near-Infrared Spectroscopy Monitoring of Renal Allograft Reperfusion in Kidney Transplant Recipients: A Feasibility and Proof-of-Concept Study
Source: J Clin Med. 2021 Sep 22;10(19):4292. doi: 10.3390/jcm10194292 (PMC8509741; doi:10.3390/jcm10194292)
Supplement: Supplementary file 1 [file jcm-10-04292-s001.zip › jcm-1373790-supplementary.pdf]

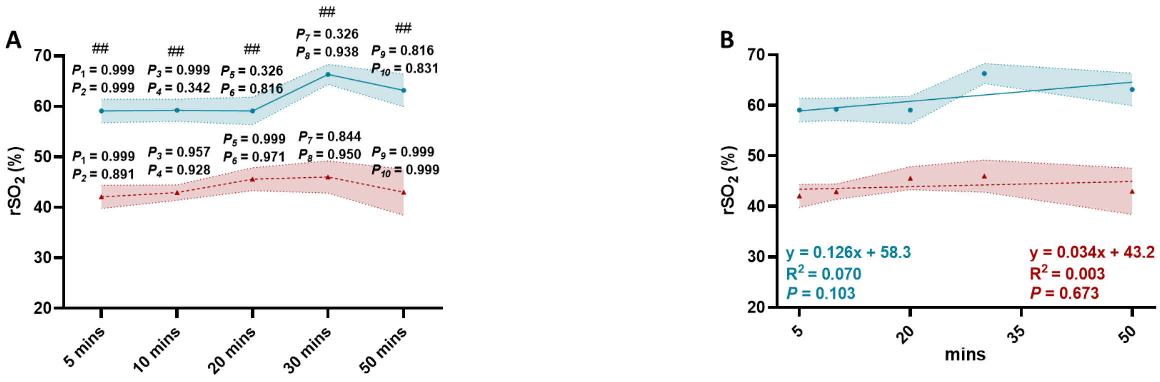

Supplementary Figure S1. Curves and linear regression analysis of average tissue oxygen saturation (rSO_2) levels measured at three regions (upper pole, renal hilum, and lower pole) of the kidney allograft in living (solid turquoise line and closed circles, LDRT) and deceased donor renal transplants (dash red line and closed triangles, DDRT) from 5 minutes to 50 minutes (mins) after reperfusion. Tissue oxygen saturation levels in living and deceased donor renal transplants were measured at 3 regions of the kidney allograft using a handheld tissue oximetry sensor device after reperfusion at 5 (n = 3 LDRT vs. 4 DDRT), 10 (n = 3 LDRT vs. 4 DDRT), 20 (n = 3 LDRT vs. 4 DDRT), 30 (n = 2 LDRT vs. 4 DDRT), and 50 minutes (n = 2 LDRT vs. 3 DDRT). Trend lines and R^2 coefficients of average tissue oxygen saturation levels measured at 3 regions of the kidney allograft from 5 mins to 50 mins after reperfusion in living and deceased donor renal transplants were calculated using linear regression analysis. A) Average tissue oxygen saturation level curves measured at 3 regions of the kidney allograft. B) Trend lines and R^2 coefficients of average tissue oxygen saturation levels measured at 3 regions of the kidney allograft. P_1: 5 mins vs. 10 mins. P_2: 5 mins vs. 20 mins. P_3: 10 mins vs. 20 mins. P_4: 10 mins vs. 30 mins. P_5: 20 mins vs. 30 mins. P_6: 20 mins vs. 50 mins. P_7: 5 mins vs. 30 mins. P_8: 30 mins vs. 50 mins. P_9: 5 mins vs. 50 mins. P_{10}: 10 mins vs. 50 mins. ##$P < .01$ vs. deceased donor kidney allografts. Trend line equations, R^2 coefficients, and P values for living and deceased donor renal transplants are displayed in matching colors. Data are expressed as mean ± SEM.
